# Efficacy and Tolerability of Erenumab and Topiramate for Prevention of Chronic Migraine: A Retrospective Cohort Study

**DOI:** 10.3390/medicina60101684

**Published:** 2024-10-14

**Authors:** Eslam El Nebrisi, Zainaba Suaad Ahmed Ruwayya, Dalya Ibrahim Alzayori, Ranya Ibrahim Alzayori, Shyam Babu Chandran, Mohamed Elshafei

**Affiliations:** 1 Department of Biomedical Sciences, Dubai Medical College for Girls, Dubai 20170, United Arab Emirates; 2 Department of Internal Medicine, Dubai Medical College for Girls, Dubai 20170, United Arab Emirates; zsa20200171@dmcg.edu (Z.S.A.R.); dim20200112@dmcg.edu (D.I.A.); rim20200143@dmcg.edu (R.I.A.); 3 Department of Neurology, Zulekha Hospital, Dubai P.O. Box 48577, United Arab Emirates; schandran@zulekhahospitals.com

**Keywords:** calcitonin gene-related peptide, Erenumab, Topiramate, migraine, prophylaxis, observational

## Abstract

*Background and Objectives:* Migraine is a chronic neurological disorder affecting approximately 14% of the global population. Beyond physical pain, migraines significantly impact individuals’ quality of life, influencing education, employment, and income levels. Topiramate, a second-generation antiepileptic medication, has demonstrated notable efficacy in reducing the occurrence of chronic migraine. Over the past three decades, extensive research has implicated the neuropeptide calcitonin gene-related peptide (CGRP) in migraine pathogenesis. Erenumab, the first FDA-approved CGRP inhibitor, received approval in 2018. This study aims to compare the clinical efficacy of Erenumab and Topiramate for migraine prevention. *Materials and Methods:* We conducted a retrospective cohort study of adults with episodic or chronic migraine over a 12-month period, comparing Erenumab (*n* = 52) and Topiramate (*n* = 56). Outcomes assessed included changes in the Migraine Disability Assessment (MIDAS) scores from baseline over the last three months of treatment and the proportion of patients achieving a ≥50% reduction in MIDAS scores by the end of the study. *Results:* The Erenumab group showed significant improvement, with nearly 79% of patients achieving a 50% reduction in their MIDAS score, with a mean reduction of 3.76. Notably, only two patients (3.8.5) discontinued treatment due to adverse events. In contrast, the Topiramate group had over 15% of patients achieve a 50% reduction in MIDAS scores, with a mean reduction of 5.89, and a had discontinuation rate of 14.2% due to adverse events. *Conclusions:* Both Topiramate and Erenumab are effective for migraine prevention. However, Topiramate has lower tolerability and more side effects, while Erenumab offers better tolerability and safety at a higher cost. Treatment decisions should be individualized based on patient needs, efficacy, safety, and cost considerations.

## 1. Introduction

Migraine is a type of cephalalgia characterized by recurring episodes of intense, pulsatile pain that can range from moderate to severe. This pain is typically localized unilaterally in the cranium and arises from the stimulation of neural fibers situated within the vascular linings of cerebral blood vessels [1,2]. In the absence of treatment, these episodes can last from four to seventy-two hours. Common associated symptoms include photophobia, phonophobia, osmophobia, nausea, and emesis [1].

Migraines pose a significant burden, affecting approximately 14% of the global population, as reported in a Global Burden of Disease study [3]. Notably, females consistently exhibit a higher prevalence across all age groups, with the ratios ranging from 2:1 to as high as 6:1 [4,5]. The highest prevalence and case count are observed in the 10–14-year age group for both genders [6].

The impact of migraines extends beyond physical pain, leading to substantial personal repercussions including effects on education, employment, and income [7]. A cross-sectional study in Austria found that about 34% of individuals with episodic or chronic headaches reported a negative impact on their professional lives, with 21.5% indicating a decline in income [8]. Furthermore, migraines affect family dynamics, with significant consequences reported by the partners and family members of patients with chronic migraines [9].

**Figure 1:**
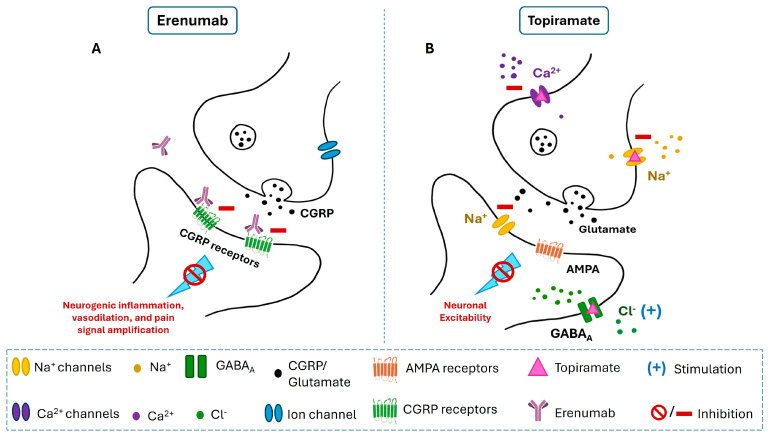
Mechanisms of action of Erenumab and Topiramate. (**A**): Erenumab is a monoclonal antibody that blocks calcitonin gene-related peptide (CGRP) receptors, preventing CGRP from binding. By inhibiting CGRP receptor activity, Erenumab reduces neurogenic inflammation, vasodilation, and pain signal amplification, which are critical pathways involved in migraine pathophysiology. (**B**): Topiramate reduces neuronal excitability through multiple actions. It blocks voltage-gated sodium (Na^+^) channels, reduces calcium (Ca^2+^) influx, and inhibits glutamate release by antagonizing AMPA receptors. Additionally, it enhances GABAergic (GABA_A_) activity, allowing more chloride (Cl^−^) ions into the neuron, thus increasing inhibitory signaling and dampening neuronal excitability.

Therefore, preventative medications are critical in alleviating the burden of this debilitating neurological condition, aiming to reduce the frequency, duration, and intensity of migraine episodes. Among various oral medications studied for migraine prevention, Topiramate, a second-generation antiepileptic drug administered at a dose of 50–100 mg per day, has demonstrated notable efficacy in reducing chronic migraine occurrence [10,11]. Its mechanism of action involves interactions with voltage-dependent sodium channels, GABA receptors, and glutamate receptors, enhancing GABA-A receptor activity while reducing glutamate activity at AMPA and kainite receptors (Figure 1). This dual mechanism effectively lowers neuronal excitability, which underlies Topiramate’s effectiveness in the prevention of migraine [12].

Over the past three decades, extensive research has implicated the neuropeptide calcitonin gene-related peptide (CGRP), the most potent known vasodilatory peptide, in the pathogenesis of migraine [13,14]. CGRP and its receptor are present in both the peripheral and central nervous systems, contributing significantly to migraine pathophysiology. CGRP affects various peripheral targets, including blood vessels, trigeminal afferents, mast cells, and glial cells in meninges, as well as neural cell bodies and satellite glia in the trigeminal ganglia [13]. Neurons along the meningeal and cerebral arteries release neuron-sensitizing agents, triggering neurogenic inflammation and stimulating mast cell release, leading to increased vasodilation in the dura. This modulation of neuronal activity is believed to initiate a feedback loop that heightens nociceptor sensitivity in the peripheral nervous system [15]. Moreover, the relatively low blood–brain barrier (BBB) permeability of CGRP-targeting monoclonal antibodies further supports the peripheral role of CGRP in migraine pathogenesis [13].

Recent advancements in migraine prevention have led to the introduction of new therapeutic options. The first calcitonin gene-related peptide (CGRP) inhibitor, Erenumab, marketed under the brand name Aimovig, was approved by the US Food and Drug Administration (FDA) in May 2018. It was approved for use as a subcutaneous injection specifically for the prevention of migraines [16]. Erenumab works by inhibiting CGRP from binding to its receptor. This action reduces migraine-related processes, thereby decreasing the frequency and severity of migraine attacks (Figure 1).

Other innovative choices include Atogepant and Rimegepant, specifically designed for episodic migraine, as well as Eptinezumab, Erenumab, Fremanezumab, and Galcanezumab, effective for both episodic and chronic migraines [10,17,18]. Monoclonal antibodies were effective in reducing migraine days by more than 50% [17,19]. Additionally, these antibodies maintain sustained efficacy for over a month after administration, allowing for monthly or quarterly prophylactic use, a distinct advantage over the daily oral administration of gepants or Topiramate [13].

This study aims to investigate and assess the clinical efficacy of Erenumab compared to Topiramate for migraine prevention, as well as to compare treatment tolerability and cost-effectiveness between the two medications.

## 2. Materials and Methods

### 2.1. Study Design

This is an observational, retrospective, cohort head-to-head comparative study conducted at the Neurology Department—Zulekha Hospital, Dubai, United Arab Emirates (UAE). The study comprised two phases: a screening phase (up to two weeks) to assess eligibility, and a data retrieval and analysis phase (five weeks). Study approval was granted by the Research and Ethics Committee at Dubai Medical College for Girls (Ref. No.: REC/DMCG/AY23-24/S-10). The study design and reporting adhered to the STROBE (Strengthening the Reporting of Observational Studies in Epidemiology) guidelines, ensuring transparent and comprehensive reporting of the findings (see Supplementary Table S1).

### 2.2. Patients and Study Groups

The study included adults aged 18–65 years with a history of episodic migraine (EM) or chronic migraine (CM) from January 2021 to December 2021 [20]. Participants had to have experienced migraines, with or without aura, for at least 12 months prior to screening, and had never received treatment with Topiramate or a CGRP-targeting monoclonal antibody. Migraine diagnosis was based on international headache society criteria 3rd edition, and migraine severity and response to medication was assessed based on the Migraine Disability Assessment Test (MIDAS) before and 3 months after starting medication [21,22]. Inclusion criteria were as follows: (1) no prior prophylactic migraine treatment; or (2) failure of up to three prior treatments due to inefficacy or intolerance, including metoprolol/propranolol, amitriptyline, and flunarizine; or (3) regular use of Topiramate or Erenumab with confirmed compliance to scheduled doses for at least 3 months. Exclusion criteria included the following: (1) migraine onset after age 50; (2) a history of cluster headaches or hemiplegic migraines; (3) inability to differentiate migraines from other headache types; and (4) prior use of valproate or botulinum toxin A for chronic migraines, as per German HTA guidelines. Additionally, the use of any migraine prophylactic medication within five half-lives or any device or procedure within one month prior to baseline and during the study was prohibited. Detailed inclusion and exclusion criteria are provided in Appendix A.

Participants were divided into two groups: Group 1 comprised 52 participants who received Erenumab, administered as a 70 mg injection once monthly, with compliance ensured by the nurses administering the injections at the clinic. Group 2 consisted of 56 participants who received Topiramate in oral tablet form, administered twice daily, with doses ranging from 50 to 100 mg per day. Compliance for Topiramate was monitored through monthly clinic visits for medication refills. After three months, the MIDAS evaluation was conducted to assess the effectiveness of both treatments. Both groups included participants from a diverse array of nationalities.

### 2.3. Variable Measurements

Patients’ Sociodemographic:

Demographic variables assessed included age (participants’ age at data collection), gender (self-reported gender as male or female), nationality (to identify potential geographic or cultural variations), and migraine disease duration (documented in months or years since diagnosis).

Treatment Outcomes—Efficacy and Tolerability:

The primary endpoint for the analysis of each medication’s efficacy was the change in MIDAS scores from baseline over the last three months of treatment. Key secondary endpoints included achieving a ≥50% reduction from baseline MIDAS scores by the end of the study (50% responder rate). Adverse events (AEs) were compared between the two groups based on the occurrence percentages recorded by the treating physician throughout the study.

### 2.4. Data Analysis Procedures

Various statistical analyses were employed to examine the data. Descriptive statistics provided insight into the central tendency and variability of MIDAS scores pre- and post-treatment. A paired samples *t*-test assessed the statistical significance of changes in MIDAS scores following treatment with Erenumab or Topiramate. Visual representations, such as bar and scatter plots, illustrated the changes in mean MIDAS scores and the correlation between pre- and post-treatment scores (Appendix B). An independent samples *t*-test compared the difference in mean scores between Erenumab and Topiramate post-treatment. The demographic diversity of the sample was also analyzed to understand the distribution across various variables. Both parametric and non-parametric tests were employed for more validity (Appendix C). All analyses were conducted using Python 3.10 and SPSS version 26.0 Windows (SPSS Inc., Armonk, NY, USA), and were throughout set at a *p*-value of less than 0.05.

## 3. Results

### 3.1. Sociodemographic Characteristics

This study included two study groups (Table 1). Group 1 included patients who received Erenumab. The sample consisted of 52 participants (*n* = 52), predominantly females (*n* = 48), with a smaller number of males (*n* = 4). The participants’ ages ranged from 18 to 60 years, with a mean age of 40.73 years (SD = 7.81). The sample represented a diverse array of nationalities, including United Arab Emirates (UAE) (*n* = 11), India (*n* = 21), Egypt (*n* = 9), Pakistan (*n* = 4), Canada/India (*n* = 1), Iran (*n* = 1), Jordan (*n* = 1), Latvia (*n* = 1), Palestine (*n* = 1), Somalia (*n* = 1), and Tanzania (*n* = 1). This diverse sample provides a comprehensive view of Erenumab’s effectiveness across different demographics.

Group 2 included patients who received Topiramate, consisting of 56 participants (*n* = 56) in total. The majority were females (*n* = 43), while the remaining were males (*n* = 13). The participants’ ages ranged from 18 to 60 years, with a mean age of 36.7 years. The sample included individuals of various nationalities, namely UAE (*n* = 4), India (*n* = 24), Egypt (*n* = 10), Pakistan (*n* = 5), Philippines (*n* = 4), Australia (*n* = 1), Ethiopia (*n* = 1), Iraq (*n* = 2), Kenya (*n* = 1), Morocco (*n* = 1), Palestine (*n* = 1), Sudan (*n* = 1), Syria (*n* = 1), and Yemen (*n* = 1). The diverse representation of nationalities in this sample contributes to a comprehensive understanding of Topiramate’s effects across different demographic groups.

### 3.2. Effects of Erenumab and Topiramate on MIDAS

In our investigation of the therapeutic efficacy of Erenumab and Topiramate in reducing migraine-induced disability, we applied the Migraine Disability Assessment (MIDAS) score as a quantifiable measure. Our patient cohort included individuals who were evaluated for their MIDAS scores both before and after the initiation of Erenumab treatment.

Table 2 presents the descriptive statistics for the MIDAS scores. Prior to the administration of Erenumab, patients had a mean MIDAS score of 15.17 (SD = 3.83), indicating a pronounced level of disability due to migraines. The dispersion of scores around this mean was moderately extensive, as evidenced by the standard deviation.

Following treatment with Erenumab, the mean MIDAS score significantly decreased to 5.79 (SD = 2.14), showcasing a substantial reduction in the level of disability. Furthermore, the standard deviation post-treatment demonstrated a contraction, signifying that the scores were more tightly clustered around the lower mean. This indicated a reduction in the variability of treatment outcomes and a more consistent response among patients. Following treatment with Topiramate, the mean MIDAS score decreased from 9.13 (SD = 3.43) to 6.20 (SD = 2.50), suggesting a significant reduction in disability among patients. This decrease in the mean MIDAS score reflects an improvement in patient outcomes. Additionally, the reduction in standard deviation indicates a narrower spread of scores around the lower mean, highlighting decreased variability in treatment outcomes and a more consistent response to the medication across the patient group. Overall, the results suggest that both Erenumab and Topiramate are effective in reducing migraine-induced disability, as evidenced by the significant reductions in MIDAS scores and the contraction of standard deviations post-treatment.

**Figure 2:**
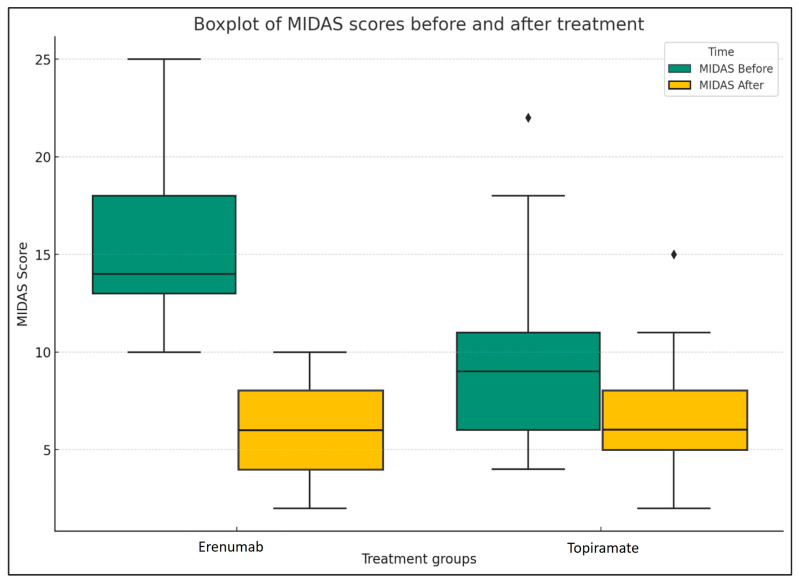
Comparison of MIDAS scores before and after treatment (Erenumab and Topiramate). The diamond-shaped (♦) points represent outliers.

Figure 2 presents a boxplot comparison of the Migraine Disability Assessment (MIDAS) scores before and after treatment for each group: Erenumab and Topiramate. The boxes represent the interquartile range (IQR) of the MIDAS scores, the line inside the box is the median, and the whiskers extend to the most extreme data points not considered outliers.

In both treatment groups, we can observe a significant decrease in MIDAS scores following treatment, which is represented by the shift from the blue to the orange boxes. This suggests that both Erenumab and Topiramate were effective in reducing the disability caused by migraines in the patient sample.

However, it is noteworthy that the Erenumab group exhibits a more substantial decrease in MIDAS scores post-treatment than the Topiramate group. This could potentially indicate that Erenumab may be more effective in reducing migraine-related disability, which is tested using the independent samples *t*-test.

An independent samples *t*-test (Table 3) was conducted to compare the MIDAS scores after treatment in the Erenumab and Topiramate groups. There was no statistically significant difference in scores for Erenumab (M = 5.79, SD = 2.14), with a standard error of the mean of 0.3, and similar results were observed for the Topiramate (M = 6.2, SD = 2.5) condition; t (94) = −0.91, *p* = 0.37. These results suggest that both treatments may have similar effects on MIDAS scores. The effect size, Cohen’s d, is −0.34. This is considered a small effect size. A negative value indicates that the mean MIDAS After score for the Erenumab group is lower than that of the Topiramate group.

Table 4 presents the results of the independent samples *t*-test. Levene’s test for equality of variances showed that the assumption of equal variances was met, as indicated by the non-significant result (F = 1.47, *p* = 0.23). The *t*-test for equality of means indicated that there was no significant difference in the MIDAS After scores between the Erenumab and Topiramate groups. The t-value was -0.91 with 106 degrees of freedom (df), resulting in a *p*-value of 0.37 (two-tailed). The mean difference between the groups was −0.408, with a standard error difference of 0.45. The 95% confidence interval for the difference ranged from −1.3 to 0.48.

**Figure 3:**
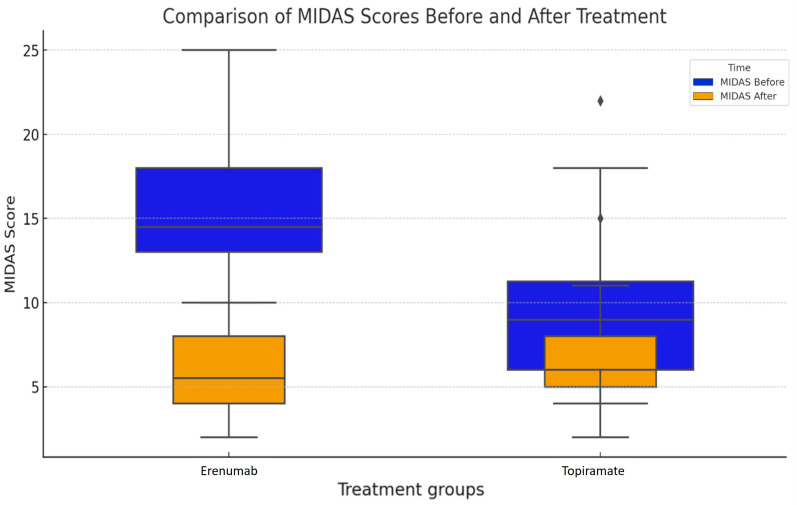
Group comparison of pre- and post-treatment. The diamond-shaped (♦) points represent outliers.

Figure 3 presents the results of an independent samples Mann–Whitney U test comparing the MIDAS scores after treatment with Erenumab and Topiramate. The histogram displays the frequency distribution of MIDAS scores for each treatment group. The Erenumab group includes 52 patients with a mean rank of 52.70, while the Topiramate group includes 56 patients with a mean rank of 56.17. The y-axis represents the MIDAS scores after treatment, and the x-axis shows the frequency of these scores within each group.

The histogram reveals the distribution patterns of MIDAS scores for both treatments, indicating that while both groups have patients with a wide range of post-treatment MIDAS scores, the Topiramate group has a slightly higher average rank, suggesting a marginal difference in the distribution of post-treatment scores between the two groups.

### 3.3. Efficacy and Tolerability

In the Topiramate group, over 15% of patients had a 50% reduction in their Migraine Disability Assessment (MIDAS) score over three months (16.07%), with a mean reduction in MIDAS of 5.89. Surprisingly, the Erenumab group demonstrated a significant reduction in migraine days compared to the Topiramate group, where nearly 79% of patients in the former group had a 50% reduction in their MIDAS score over three months (78.85%), with a mean reduction in MIDAS of 3.76 (Table 5).

In our study, the most common adverse events in Topiramate patients were cognitive impairment, nausea, weight loss, and paresthesia, with 14.20% discontinuing the medicine due to side effects. In the Erenumab group, two patients discontinued treatment due to worsening hypertension (Table 6).

## 4. Discussion

Both Erenumab and Topiramate are approved globally as preventive treatments for migraines, albeit through different mechanisms of action. Topiramate, an antiepileptic drug, enhances gamma-aminobutyric acid (GABA) activity and inhibits excitatory neurotransmitters [11]. In contrast, Erenumab is a human IgG2 monoclonal CGRP receptor–blocking antibody that specifically targets the calcitonin gene-related peptide (CGRP) receptor, blocking its interaction with CGRP, a key player in migraine onset [13]. Our study assessed the differences in efficacy, safety, tolerability, and cost-effectiveness of these two medications.

Both Erenumab and Topiramate have demonstrated efficacy in clinical trials for reducing the frequency and severity of migraine attacks; however, direct head-to-head comparisons are limited. In a double-blind, randomized, placebo-controlled trial conducted by Diener et al. (2007), Topiramate showed a significant decrease in migraine frequency, achieving an average reduction of 3.3 migraine days per month [23]. Silberstein et al. (2004) also reported a meaningful reduction with Topiramate, averaging 2.5 migraine days per month [11].

Erenumab has similarly demonstrated efficacy in reducing migraine frequency. The ARISE trial showed that Erenumab significantly decreased migraine days in patients with episodic migraine, with an average reduction of 2.9 migraine days per month [24]. Furthermore, in a phase 3b study by Reuter et al. (2018), Erenumab proved effective in patients for whom two to four prior preventive treatments had failed [25]. Our study found that nearly 79% of Erenumab patients achieved a 50% reduction in MIDAS scores over three months, with a mean reduction in MIDAS of 9.3.

Comparison of the two medications revealed a significant difference in efficacy, with Erenumab outperforming Topiramate. This discrepancy may be due to the lower doses of Topiramate used in our study (50–100 mg daily) owing to tolerability issues, as well as the lower baseline MIDAS scores in the Topiramate group (15.1 vs. 9.1).

Topiramate is associated with several adverse events, including cognitive impairment, paresthesia, weight loss, and mood changes. In the PROMPT trial, common adverse events included paresthesia (23.5%), fatigue (12.6%), and cognitive disorder (9.7%) [23]. Additionally, Topiramate carries teratogenic risks and is contraindicated during pregnancy. In our study, the most common adverse events among Topiramate patients were cognitive impairment, weight loss, and paresthesia, leading to a 14.20% discontinuing rate due to side effects. Recent safety concerns have also emerged regarding Topiramate’s use in pregnancy, prompting new guidelines to mitigate risks of neurodevelopmental issues and birth defects [26]. Furthermore, the medication poses risks of kidney stones due to metabolic acidosis, and renal complications linked to chronic kidney disease [27,28,29]. Additionally, cases of dysgeusia, including carbonation and sweet taste perversion, have been reported with the use of Topiramate in migraine prevention. Such taste disturbances can negatively affect patients’ quality of life and lead to reduced therapy adherence [30].

In contrast, Erenumab exhibited a favorable safety and tolerability profile. The ARISE trial reported that common adverse events included injection site reactions (3.3% for Erenumab vs. 2.5% for placebo) and constipation (2.7% for Erenumab vs. 1.4% for placebo) [24]. Similar side effects were also reported in a long-term study that confirmed Erenumab’s sustained efficacy in reducing migraine days, while reporting minimal discontinuations due to side effects, solidifying its role as a long-term preventive treatment option [31]. Notably, no patients in the Erenumab group discontinued treatment due to adverse events. Our findings echoed these results: 3.8% of patients experienced constipation, and 3.8% had elevated blood pressure, with two patients discontinuing treatment due to worsening hypertension. A recent study by Muñoz-Vendrell et al. (2023) highlighted mild injection site reactions and a slight increase in blood pressure, reinforcing Erenumab’s overall tolerability while suggesting caution for patients with pre-existing hypertension [32]. Cost and access to treatment are crucial considerations when selecting the appropriate medication. While Topiramate is available in a generic form and is relatively inexpensive, Erenumab is a newer biological agent that is more costly and requires special authorization from health insurance companies. Despite its higher price and limited accessibility, Erenumab may prove more cost-effective in the long term due to its superior efficacy and tolerability, which could enhance adherence and reduce healthcare utilization. Research by Buse et al. (2018) indicated that patients treated with Erenumab experienced significant improvements in migraine-related disability, quality of life, and treatment satisfaction compared to those treated with a placebo [33]. In our study, Erenumab showed better tolerability and adherence over three months, with discontinuation rates of 3.8% vs. 16.6% in the Topiramate group.

A 10-year longitudinal meta-analysis evaluated the clinical effectiveness of migraine treatments, including Topiramate and Erenumab, consistently demonstrating better safety and tolerability profiles for Erenumab. While Topiramate is effective, it is associated with more side effects, particularly cognitive issues. Supporting studies highlighted Erenumab’s efficacy, especially for patients intolerant to other treatments [34]. Despite its higher cost, Naghdi et al. suggested that Erenumab’s benefits could justify its expenses through improved patient outcomes [35]. However, real-world barriers, such as insurance challenges, emphasize the need for long-term validation of these treatments across diverse populations [36].

Our study was conducted on a multicultural population, enhancing the applicability of our findings across diverse sociodemographic groups. The results align with those of other studies, further confirming the safety and efficacy of both medications in treating chronic migraine.

However, we acknowledge several limitations of our study. There are few randomized controlled trials (RCTs) directly comparing Topiramate and Erenumab, and our retrospective design is susceptible to recall bias. Additionally, the limited sample size restricts definitive conclusions, particularly regarding medication tolerability. A multicenter RCT could provide a more robust comparison of first-line therapies.

Future research should explore higher doses of Erenumab (140 mg), use larger sample sizes, and extend follow-up periods beyond one year to gain comprehensive insights into the long-term efficacy and safety of these treatments. Such efforts would enhance the generalizability of our findings to a broader population over an extended timeframe.

To the best of our knowledge, this study is the first to compare Erenumab and Topiramate in the UAE population. Although the FDA approved Erenumab in 2018 for its anti-migraine effects, it is not currently considered a first-line therapy in the UAE. Patients must demonstrate the failure of at least two traditional prophylactic treatments before insurance approval for Erenumab, which involves multiple authorization steps and can delay treatment. This context underscores the novelty of our study, addressing real-world challenges and the comparative effectiveness of these treatments within the UAE healthcare system.

## 5. Conclusions

This study is the first to directly compare the efficacy, safety, and cost-effectiveness of Topiramate and Erenumab for migraine prevention in the UAE population. Erenumab demonstrated superior safety and tolerability, making it a preferable option for patients sensitive to medication side effects, while Topiramate remains a widely available and cost-effective treatment. However, the cost of Erenumab is a significant consideration, particularly in the short term, despite its favorable safety profile. When selecting between these two treatments, clinicians must balance the benefits of efficacy and safety with the financial implications for patients. The findings from this study highlight the necessity of individualized treatment decisions, ensuring that therapy choices are tailored to each patient’s clinical needs, risk factors, and financial circumstances. Future research should focus on the long-term outcomes of these treatments, particularly in larger and more diverse populations, to further inform clinical decision-making.

Clinical Implications:

- Both medications are effective in migraine prevention.
- Topiramate has lower tolerability due to its daily oral dosage and potential side effects.
- Erenumab offers better tolerability as a monthly injection with a more favorable safety profile.
- Erenumab has a higher monthly cost compared to Topiramate.
- The choice between Topiramate and Erenumab should be individualized, considering factors such as efficacy, safety, tolerability, convenience, and cost.
- Shared decision-making with patients is essential to select the most suitable treatment for effective migraine management, minimizing adverse effects, and optimizing quality of life.

## Figures and Tables

**Table 1:** Erenumab and Topiramate sociodemographic variables.

		Erenumab	Topiramate
**Age**	Mean	40.7	36.7
**Sex**	Female	48	43
	Male	4	13
**Nationality**	UAE	11	4
	India	21	24
	Egypt	9	10
	Pakistan	4	5
	Philippines	-	4
	Australia	-	1
	Canada/India	1	-
	Ethiopia	-	1
	Iran	1	-
	Iraq	-	1
	Jordan	1	-
	Kenya	-	1
	Latvia	1	-
	Morocco	-	1
	Palestine	1	1
	Somalia	1	-
	Sudan	-	1
	Syria	-	1
	Tanzania	1	-
	Yemen	-	1
Total		52	56

**Table 2:** Baseline MIDAS score before and after treatment.

Comparative Groups		Mean	*n*	SD	Std. Error Mean
**Erenumab**	MIDAS Before (Erenumab)	15.17	52	3.83	0.53
	MIDAS After (Erenumab)	5.79	52	2.14	0.3
**Topiramate**	MIDAS Before (Topiramate)	9.13	56	3.43	0.46
	MIDAS After (Topiramate)	6.20	56	2.50	0.33

**Table 3:** Group statistics for MIDAS scores after treatment with Erenumab and Topiramate.

	Comparative Groups	*n*	Mean	SD	Std. Error Mean
**MIDAS After**	Erenumab	52	5.79	2.14	0.3
	Topiramate	56	6.2	2.5	0.33

**Table 4:** Independent samples *t*-test results for MIDAS After scores between Erenumab and Topiramate groups.

MIDAS after Equal Variances Assumed Comparative Groups	Levene’s Test for Equality of Variances		*t*-Test for Equality of Means						
	F	Sig.	t	df	*p*-Value	Mean Difference	Std. Error Difference	95% Confidence Interval of the Difference	
								Lower	Upper
**Erenumab**	1.47	0.23	−0.91	106	0.37	−0.41	0.45	−1.3	0.48
**Topiramate**			−0.92	105.31	0.36	−0.41	0.45	−1.3	0.48

**Table 5:** Efficacy and Tolerability of Aimovig and Topamax in the last 3 months of the treatment.

	Efficacy							Tolerability
Comparative Groups		*n*	Mean	Skewness		Kurtosis		
				Statistics	Standard Error	Statistics	Standard Error	
**Erenumab**	A 50% reduction in MIDAS scores in the last 3 months	52	3.76	1.275	0.33	0.41	0.65	100%
**Topiramate**	A 50% reduction in MIDAS scores in the last 3 months	56	5.89	0.52	0.32	0.56	0.63	14.02%

**Table 6:** Side effects of Topiramate and Erenumab.

Group	Patients with >50% Reduction in MIDAS Score (%)	Total Patients in Group	Number of Patients with >50% Reduction	Patients Discontinued Due to Side Effects (%)	Side Effects
**Erenumab**	79%	52	41	3.8% (2/52)	Hypertension (2 patients), Constipation (2 patients)
**Topiramate**	16.6%	56	9	14.2% (8/56)	Cognitive disability, paresthesia, nausea (8 patients)

**Figure A1:**
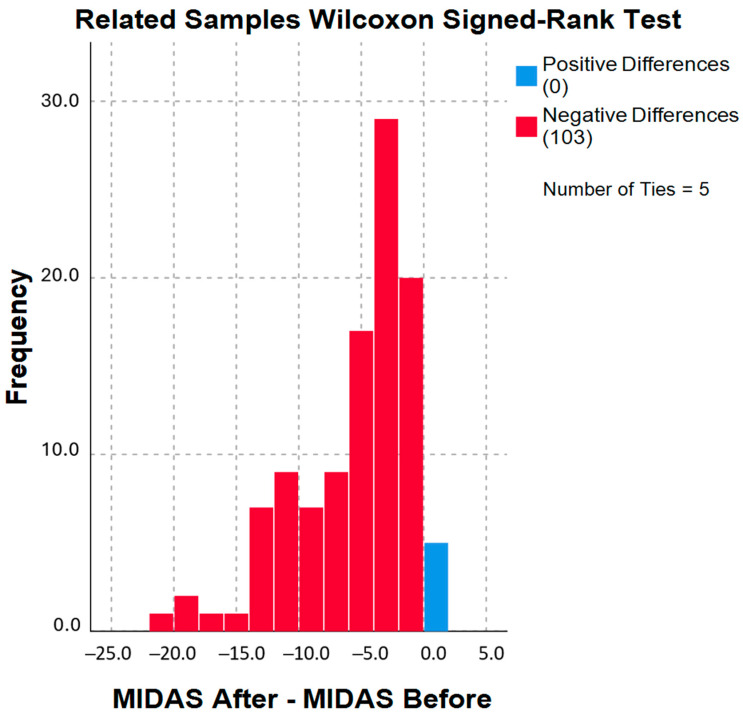
Related samples Wilcoxon signed-rank test for MIDAS scores.

**Figure A2:**
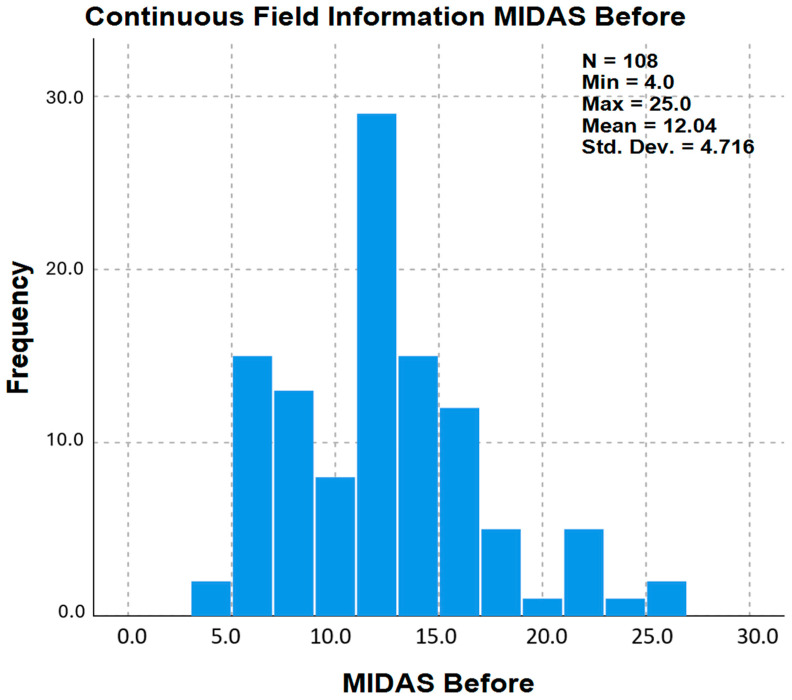
Distribution of MIDAS scores before intervention.

**Figure A3:**
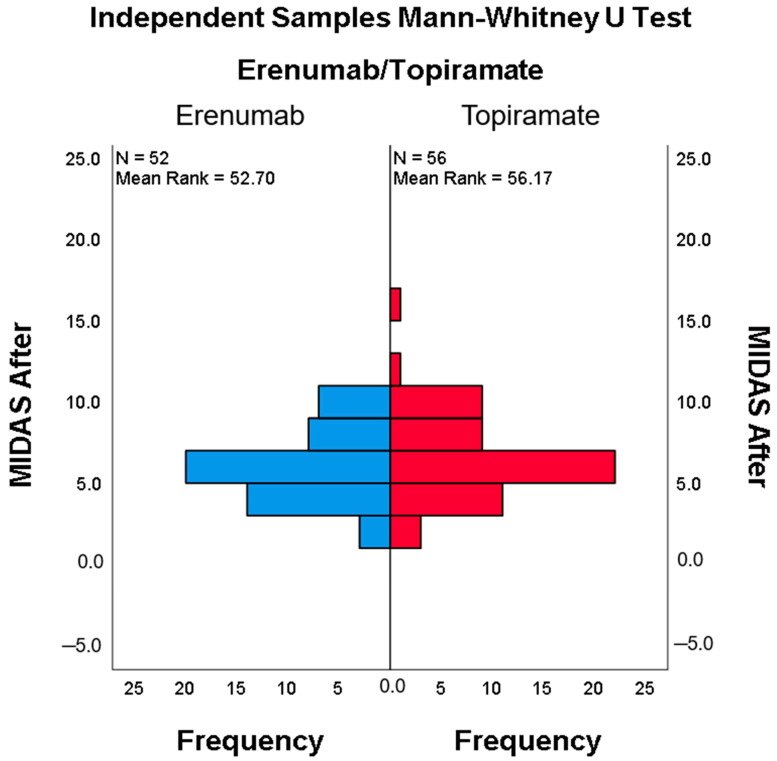
MIDAS scores post-treatment with Erenumab and Topiramate: independent samples Mann–Whitney U test.

## Data Availability

The original contributions presented in the study are included in the article/Supplementary Materials. Further inquiries can be directed to the corresponding authors.

## Supplementary Materials

The following supporting information can be downloaded at https://www.mdpi.com/article/10.3390/medicina60101684/s1, Table S1: STROBE Statement—checklist of items that should be included in reports of observational studies.

## Appendix A

**Inclusion and Exclusion Criteria**

**Inclusion criteria:**

- Patients were eligible if they were treatment-naive to prophylactic migraine therapies or had not responded adequately or tolerated up to three prior preventive treatments, which could include metoprolol/propranolol, amitriptyline, or flunarizine.

Exclusion criteria:

- Patients were excluded if they experienced their first migraine after the age of 50, had a history of cluster headaches or hemiplegic migraines, or could not distinguish migraines from other types of headaches.
- Additionally, patients were excluded if they had previously been treated with valproate or, in cases of chronic migraine, with botulinum toxin A, as per the recommendations of the German HTA bodies.

## Appendix B

**Parametric Tests**

**Table A1:** Paired samples correlations.

Comparative Groups		*n*	Correlation	*p*-Value
**Erenumab**	MIDAS Before Erenumab and MIDAS After Erenumab	52	−0.043	0.760
**Topiramate**	MIDAS Before Topiramate and MIDAS After Topiramate	56	0.78	0.000

## Appendix C

**Non-Parametric Tests**

**Table A2:** Hypothesis test summary for MIDAS score differences before and after treatment.

	Null Hypothesis	Test	Sig.	Decision
1	The median of differences between MIDAS Before and MIDAS After equals 0.	Related Samples Wilcoxon Signed-Rank Test	0.000	Reject the null hypothesis *

* Asymptotic significances are displayed. The significance level is ≤0.05.

**Table A3:** Related samples Wilcoxon signed-rank test summary.

Total *n*	108
Test Statistic	0.000
Standard Error	303.555
Standardized Test Statistic	−8.822
Asymptotic Sig. (2-sided test)	0.000

The histogram illustrates the frequency distribution of differences in MIDAS scores before and after the intervention. The data were analyzed using the Wilcoxon signed-rank test. The x-axis represents the difference in MIDAS scores (MIDAS After–MIDAS Before), while the y-axis indicates the frequency of each difference value.

This histogram shows the distribution of MIDAS scores for all participants before intervention. The MIDAS scores range from 4 to 25 with a mean score of 12.04 and a standard deviation of 4.716.

The histogram in Figure A2 represents the frequency distribution of MIDAS scores before any intervention. The MIDAS score, which ranges from 4 to 25 in this sample, is a measure of migraine-related disability. The x-axis represents the MIDAS scores, and the y-axis represents the frequency of each score within the sample. The descriptive statistics shown on the graph indicate that the sample size (N) is 108, with a minimum score of 4.0, a maximum score of 25.0, a mean score of 12.04, and a standard deviation of 4.716. The histogram shows that most scores are clustered between 10 and 15, indicating that a significant portion of the sample experiences moderate to high levels of migraine-related disability.

**Table A4:** Hypothesis test summary for MIDAS score distribution across Erenumab and Topiramate treatment groups.

	Null Hypothesis	Test	Sig.	Decision
1	The distribution of MIDAS After is the same across categories of Erenumab/Topiramate.	Independent Samples Mann–Whitney U Test	0.560	Retain the null hypothesis *

* Asymptotic significances are displayed. The significance level is 0.050.

**Table A5:** Independent samples Mann–Whitney U test summary.

Total *n*	108
Mann–Whitney U	1549.500
Wilcoxon W	3145.500
Test Statistic	1549.500
Standard Error	160.262
Standardized Test Statistic	0.583
Asymptotic Sig. (2-sided test)	0.560
