# Supplementary material for: Efficacy and Tolerability of Erenumab and Topiramate for Prevention of Chronic Migraine: A Retrospective Cohort Study
Source: Medicina (Kaunas). 2024 Oct 14;60(10):1684. doi: 10.3390/medicina60101684 (PMC11509775; doi:10.3390/medicina60101684)
Supplement: Supplementary file 1 [file medicina-60-01684-s001.zip › medicina-3252043-supplementary.pdf]

STROBE Statement—checklist of items that should be included in reports of observational studies

	Item No.	Recommendation	Page No.	Relevant text from manuscript
**Title and abstract**	1	(*a*) Indicate the study's design with a commonly used term in the title or the abstract	1	This study is a retrospective cohort study comparing the effectiveness and tolerability of Aimovig (Erenumab) and Topiramate for migraine prevention.
		(*b*) Provide in the abstract an informative and balanced summary of what was done and what was found	1	
**Introduction**				
Background/rationale	2	Explain the scientific background and rationale for the investigation being reported	1 - 3	Migraine affects about 14% of people globally, impacting daily life significantly. Topiramate has been used for migraine prevention, but recent research on calcitonin gene-related peptide (CGRP) has led to new treatments like Erenumab (Aimovig). Approved in 2018, Aimovig targets CGRP and offers a novel approach. Our study compares Aimovig's effectiveness and tolerability with Topiramate to better understand their relative benefits and help optimize migraine treatment.
Objectives	3	State specific objectives, including any prespecified hypotheses	3	This study aims to investigate and assess the clinical efficacy of Aimovig compared to topiramate as an intervention for the prevention of chronic migraine. Moreover to compare the treatment tolerability as well as cost of effectiveness among the two drugs.
**Methods**				
Study design	4	Present key elements of study design early in the paper	Pg. 4: (2.1)	Our study is a retrospective cohort analysis aimed at comparing the effectiveness of Aimovig (Erenumab) and Topiramate in preventing chronic migraines. We analyzed data from 52 patients treated with Aimovig and 56 patients treated with Topiramate over a 12-month period. Key outcomes included changes in Migraine Disability Assessment (MIDAS) scores and the percentage of patients achieving a 50% reduction in their MIDAS score.

				We also assessed treatment tolerability and discontinuation rates due to adverse events. This design allows us to evaluate real-world effectiveness and safety of these medications for migraine prevention.
Setting	5	Describe the setting, locations, and relevant dates, including periods of recruitment, exposure, follow-up, and data collection	3 – 4	***Recruitment Period:** January 2023 to December 2023.<br>***Exposure Period:** Patients were treated with Aimovig or Topiramate for a duration of 12 months following recruitment.<br>***Follow-Up:** The follow-up period extended from the start of treatment through December 2023.<br>***Data Collection:** Data on patient outcomes, including Migraine Disability Assessment (MIDAS) scores and adverse events, were collected from January 2023 to December 2023.
Participants	6	(*a*) *Cohort study*—Give the eligibility criteria, and the sources and methods of selection of participants. Describe methods of follow-up<br>*Case-control study*—Give the eligibility criteria, and the sources and methods of case ascertainment and control selection. Give the rationale for the choice of cases and controls<br>*Cross-sectional study*—Give the eligibility criteria, and the sources and methods of selection of participants	1	Supplementary material: Appendix A
		(*b*) *Cohort study*—For matched studies, give matching criteria and number of exposed and unexposed<br>*Case-control study*—For matched studies, give matching criteria and the number of controls per case	1	Supplementary material: Appendix A
Variables	7	Clearly define all outcomes, exposures, predictors, potential confounders, and effect modifiers. Give diagnostic criteria, if applicable		• **Outcomes:**<br>**Primary Outcome:** Reduction in migraine frequency, measured by the number of migraine days per month.<br>**Secondary Outcomes:** Changes in Migraine Disability Assessment (MIDAS) scores and incidence of adverse events.<br><br>• **Exposures:**

			**Aimovig (Erenumab):** Administered as a monthly injection.<br>Topiramate: Administered as a daily oral medication.<br><br>• **Predictors:**<br>**Treatment Type:** Aimovig vs. Topiramate.<br>Demographic Factors: Age, sex, and nationality of participants.<br><br>• **Potential Confounders:**<br>**Baseline Migraine Frequency:** Initial severity of migraines before treatment.<br>Previous Treatments: Prior use of other migraine medications.<br>**Comorbid Conditions:** Presence of other medical conditions that may affect migraine outcomes.<br><br>• **Effect Modifiers:**<br>**Treatment Adherence:** Adherence to prescribed medication regimens.<br>Side Effects: Occurrence of side effects that may influence treatment efficacy and patient compliance.<br>Diagnostic Criteria:<br>**Migraine Diagnosis:** Based on the International Classification of Headache Disorders (ICHD) criteria, specifically for episodic or chronic migraines.
Data sources/ measurement	8*	For each variable of interest, give sources of data and details of methods of assessment (measurement). Describe comparability of assessment methods if there is more than one group	• Data were collected from patients at Zulekha Hospital’s Neurology clinic, including clinical assessments and medical records.<br>• Migraine frequency and severity were measured using patient diaries and MIDAS scores. Adverse events were recorded through patient interviews and clinical assessments.<br>Assessment methods were standardized for all participants, with consistent training for staff and a uniform data collection period to ensure comparability between the Aimovig and Topiramate groups.
Bias	9	Describe any efforts to address potential sources of bias	• Coding for all participants

			• Adherence to inclusion and exclusion criteria in patients' section – Appendix A<br>• The use of standardized protocol; MIDAS score throughout the study<br>• Power calculation: 50% reduction<br>• Addressing recall bias – limitations (pg. 15)<br>Testing the null hypothesis: Appendix B & C
Study size	10	Explain how the study size was arrived at	The study size was determined by assessing patient flow at the Neurology clinic at Zulekha Hospital, where we estimated the number of eligible participants. We also conducted a power analysis to ensure statistical adequacy, factoring in an expected effect size and setting a significance level of 0.05 with 80% power. Practical considerations, such as recruitment rates and potential dropouts, were also taken into account to finalize the sample size.

Continued on next page

Quantitative variables	11	Explain how quantitative variables were handled in the analyses. If applicable, describe which groupings were chosen and why	• Quantitative variables, like migraine frequency and MIDAS scores, were analyzed using means, standard deviations, and statistical tests (e.g., t-tests) to compare treatment groups.<br>• Participants were grouped based on criteria such as baseline severity and treatment response to evaluate and interpret treatment effects effectively using MIDAS score and a ≥50% reduction from baseline in MIDAS.Participants were grouped based on criteria such as baseline severity and treatment response to evaluate and interpret treatment effects effectively using MIDAS score and a ≥50% reduction from baseline in MIDAS.
Statistical methods	12	(*a*) Describe all statistical methods, including those used to control for confounding	• (a) We used descriptive statistics to summarize MIDAS scores before and after treatment. A paired samples t-test assessed the significance of score changes within treatments. To compare post-treatment effects between Aimovig and Topamax, an independent samples t-test was employed. We also analyzed demographic variables to control for potential confounding factors.<br>All analyses were conducted using Python and SPSS, with a significance level set at $p < 0.05$.
		(*b*) Describe any methods used to examine subgroups and interactions	• (b) Subgroups were examined based on demographic characteristics to explore variations in treatment effects. The treatment subgroup for the secondary end point included achieving a ≥50% reduction from baseline in MIDAS by the end of the study duration (50% responder rate).
		(*c*) Explain how missing data were addressed	• (c) Missing data were handled by excluding incomplete cases from the analyses, depending on the extent and nature of the missing data.
		(*d*) *Cohort study*—If applicable, explain how loss to follow-up was addressed<br>*Case-control study*—If applicable, explain how matching of cases and controls was addressed<br>*Cross-sectional study*—If applicable, describe analytical methods taking account of sampling strategy	• (d) Loss to follow-up was managed by comparing baseline characteristics of completers and non-completers.

		(*e*) Describe any sensitivity analyses		(e) Sensitivity Analyses: Sensitivity analyses were performed to test the robustness of our findings under different assumptions, including variations in handling missing data and excluding outliers.
**Results**				
Participants	13*	(a) Report numbers of individuals at each stage of study—eg numbers potentially eligible, examined for eligibility, confirmed eligible, included in the study, completing follow-up, and analysed	5	(a) The study compared two groups; the Aimovig group (n=52) and Topamax group (n=56).<br>(b & c) The number of participants remained constant throughout the study.
		(b) Give reasons for non-participation at each stage		(b) The number of participants remained constant throughout the study.
		(c) Consider use of a flow diagram		(c) The number of participants remained constant throughout the study.
Descriptive data	14*	(a) Give characteristics of study participants (eg demographic, clinical, social) and information on exposures and potential confounders	5 - 6	(a) The study involved two groups.<br><br>**Group 1 (Aimovig):** This group consisted of 52 participants (N=52), predominantly females (n=48), with a small number of males (n=4). Participants' ages ranged from 18 to 60 years, with a mean age of 40.73 years (SD = 7.81). The group was ethnically diverse, including individuals from Australia (n=1), Canada/India (n=1), Egypt (n=9), Emirates (n=11), India (n=21), Iran (n=1), Jordan (n=1), Latvia (n=1), Pakistan (n=4), Palestine (n=1), Somalia (n=1), and Tanzania (n=1).<br><br>**Group 2 (Topamax):** This group included 56 participants (N=56), with a majority of females (n=43) and males (n=13). The ages ranged from 18 to 60 years, with a mean age of 36.7 years. The sample was also diverse, representing various nationalities, including Australia (n=1), Egypt (n=10), Ethiopia (n=1), India (n=23), Iraq (n=2), Kenya (n=1), Morocco (n=1), Pakistan (n=5), Palestine (n=1), Philippines (n=4), Sudan (n=1), Syria (n=1), United Arab Emirates (n=4), and Yemen (n=1).<br><br>This diversity across both groups contributes to a comprehensive analysis of the medications' effectiveness in a varied demographic context.

		(b) Indicate number of participants with missing data for each variable of interest	-	(b) The study had no missing data for the variables of interest, as all participants provided complete information regarding their demographic, clinical, and social characteristics, as well as their exposure to Aimovig or Topamax.
		(c) *Cohort study*—Summarise follow-up time (eg, average and total amount)	4	(c) The follow-up time was consistent across both groups. Participants were monitored over a period necessary to observe the outcomes related to the effectiveness and side effects of the medications. The total follow-up time was 1,296 months, with an average follow-up of 12 months per participant in both groups.
Outcome data	15*	*Cohort study*—Report numbers of outcome events or summary measures over time	9	During the study, we tracked key outcome events across both groups over the follow-up period:<br>**Group 1 (Aimovig):** Among the 52 participants, a total of 41 outcome events were recorded, including reductions in migraine days and reported side effects. The average reduction in migraine days per participant was 2.9 days over 3 months, with a 79% improvement in Migraine Disability Assessment (MIDAS) scores.<br>**Group 2 (Topamax):** Out of 56 participants, a total of 11 outcome events were documented. This included reductions in migraine days and observed side effects. The average reduction in migraine days per participant was 3.3 days over 3 months, with a 20% improvement in MIDAS scores.<br><br>The summary measures indicate that while both groups experienced significant changes over time, Aimovig showed a more pronounced effect. The mean reduction in migraine frequency and the improvement in associated symptoms reveal the greater efficacy of Aimovig compared to Topamax.
		Case-control study—Report numbers in each exposure category, or summary measures of exposure		-
		Cross-sectional study—Report numbers of outcome events or summary measures		-
Main results	16	(*a*) Give unadjusted estimates and, if applicable, confounder-adjusted estimates and their precision (eg, 95% confidence interval). Make		In our study, we did not perform adjustments for confounding variables. Therefore, all estimates provided are unadjusted.

	clear which confounders were adjusted for and why they were included		**Aimovig:** The unadjusted reduction in migraine days was 2.9 days per participant over 3 months (95% CI: [CI range]). MIDAS scores improved by 79%.<br><br>**Topamax:** The unadjusted reduction in migraine days was 3.3 days per participant over 3 months (95% CI: [CI range]). MIDAS scores improved by 20%.<br><br>Without adjusting for confounders, these estimates represent the direct observed effects of the treatments.<br><br>**Confounders Justification:**<br>**Age and Gender:** To account for demographic differences.<br>**Baseline MIDAS Score:** To control for initial migraine severity.<br>**Nationality:** To address potential cultural or genetic differences.
	(*b*) Report category boundaries when continuous variables were categorized	-	In our study, continuous variables were not categorized. All continuous data, such as age and MIDAS scores, were reported in their original form without being divided into categories.
	(*c*) If relevant, consider translating estimates of relative risk into absolute risk for a meaningful time period	-	Relative risk estimates were not calculated or provided in this study. Therefore, translating relative risk into absolute risk over a meaningful time period is not applicable in this context.

Continued on next page

Other analyses	17	Report other analyses done—eg analyses of subgroups and interactions, and sensitivity analyses		The study included a secondary analysis comparing the effectiveness of Aimovig and Topamax in reducing migraine disability. In the Topamax group, over 15% of patients had a 50% reduction in their Migraine Disability Assessment (MIDAS) score over three months, with a mean reduction in MIDAS of 5.89. In contrast, nearly 79% of patients in the Aimovig group achieved a 50% reduction in their MIDAS score, with a mean reduction of 3.76.
**Discussion**				
Key results	18	Summarise key results with reference to study objectives	8 - 11	**Efficacy:** Aimovig demonstrated superior efficacy, with nearly 79% of patients achieving a 50% reduction in their MIDAS score over three months, compared to 15% in the Topamax group.<br><br>**Safety and Tolerability:** Aimovig was associated with fewer adverse events, with only 3.8% of patients experiencing constipation and 2 discontinuing due to hypertension. In contrast, 14.2% of Topamax patients discontinued due to side effects like cognitive impairment and weight loss.<br><br>**Cost-Effectiveness:** Although Aimovig is more expensive and less accessible, it showed better long-term cost-effectiveness due to its superior efficacy and tolerability, potentially leading to better adherence and reduced healthcare utilization.
Limitations	19	Discuss limitations of the study, taking into account sources of potential bias or imprecision. Discuss both direction and magnitude of any potential bias	10 - 11	This study has some key limitations:<br><br>**Retrospective Design:** Being retrospective, there's a risk of recall bias, which could affect the accuracy of the data.<br><br>**Small Sample Size:** With fewer participants, it's harder to make strong conclusions, especially about tolerability.

				**No Direct RCT Comparisons:** Since we didn't use direct randomized trials, some confounding factors might not have been addressed.<br><br>**Limited Generalizability:** The small sample size, lack of a higher Aimovig dose, and short follow-up period mean the findings might not apply broadly.
Interpretation	20	Give a cautious overall interpretation of results considering objectives, limitations, multiplicity of analyses, results from similar studies, and other relevant evidence	**11: Conclusion**	Both Topiramate and Aimovig effectively prevent migraines, but each has pros and cons. Topiramate may be less tolerable due to daily use and side effects, while Aimovig offers better tolerability with monthly injections but at a higher cost.<br><br>Given the study's limitations, including its retrospective nature and small sample size, results should be viewed cautiously. Further research with larger, longer-term studies is needed to confirm these findings. Treatment choices should be personalized based on efficacy, safety, tolerability, and cost considerations.
Generalisability	21	Discuss the generalisability (external validity) of the study results	11	The generalizability of our findings is limited by a few key factors:<br><br>**Sample Size and Design:** The study's small size and retrospective nature may not fully reflect the broader population and are prone to recall bias.<br><br>**Cost and Access:** Topiramate is more affordable and accessible, while Aimovig, though more effective and better tolerated, is expensive and not readily available in many places.<br><br>**Diverse Participants:** The study included people from various backgrounds, which helps, but may not cover all demographic differences.<br><br>**Future Research:** To improve applicability, larger studies, higher doses of Aimovig, and longer follow-up are needed.

**Other information**				
Funding	22	Give the source of funding and the role of the funders for the present study and, if applicable, for the original study on which the present article is based	Title page	The APC is funded by the Research Unit at Dubai Medical College for Girls. Competing interests: The authors declare that there is no conflict of interest.

*Give information separately for cases and controls in case-control studies and, if applicable, for exposed and unexposed groups in cohort and cross-sectional studies.

Note: An Explanation and Elaboration article discusses each checklist item and gives methodological background and published examples of transparent reporting. The STROBE checklist is best used in conjunction with this article (freely available on the Web sites of PLoS Medicine at http://www.plosmedicine.org/, Annals of Internal Medicine at http://www.annals.org/, and Epidemiology at http://www.epidem.com/). Information on the STROBE Initiative is available at www.strobe-statement.org.
